# Bad habits–good goals? Meta-analysis and translation of the habit construct to alcoholism

**DOI:** 10.1038/s41398-024-02965-1

**Published:** 2024-07-19

**Authors:** F. Giannone, C. Ebrahimi, T. Endrass, A. C. Hansson, F. Schlagenhauf, W. H. Sommer

**Affiliations:** 1 Institute of Psychopharmacology, Central Institute of Mental Health, Medical Faculty Mannheim, Heidelberg University, 68159 Mannheim, Germany; 2 Faculty of Psychology, Institute of Clinical Psychology and Psychotherapy, Technische Universität Dresden, 01062 Dresden, Germany; 3 Department of Psychotherapy, Campus Charité Mitte, Charité Universitätsmedizin Berlin & St. Hedwig Hospital, 10117 Berlin, Germany; 4 Bethania Hospital for Psychiatry, Psychosomatics and Psychotherapy, Greifswald, Germany; 5 German Center for Mental Health (DZPG), Partner Site Mannheim-Heidelberg-Ulm, 68159 Mannheim, Germany

**Keywords:** Neuroscience, Diseases

## Abstract

Excessive alcohol consumption remains a global public health crisis, with millions suffering from alcohol use disorder (AUD, or simply “alcoholism”), leading to significantly reduced life expectancy. This review examines the interplay between habitual and goal-directed behaviors and the associated neurobiological changes induced by chronic alcohol exposure. Contrary to a strict habit-goal dichotomy, our meta-analysis of the published animal experiments combined with a review of human studies reveals a nuanced transition between these behavioral control systems, emphasizing the need for refined terminology to capture the probabilistic nature of decision biases in individuals with a history of chronic alcohol exposure. Furthermore, we distinguish habitual responding from compulsivity, viewing them as separate entities with diverse roles throughout the stages of the addiction cycle. By addressing species-specific differences and translational challenges in habit research, we provide insights to enhance future investigations and inform strategies for combatting AUD.

## Introduction

‘Old habits die hard’—this folklore seems to aptly describe the challenges faced in addiction therapy. Furthermore, colloquial language often equates addiction with ‘bad’ habits. Even as early as the original edition of Webster’s Dictionary in 1828 [1], it was written: “Frequent drinking of spirits leads to a *habit* of intemperance. We should endeavor to correct evil habits by a change of practice.” Throughout history, alcohol consumption has been accompanied by individual tragedies and public health disasters, contributing to its controversial and sometimes hypocritical portrayal [2].

Currently, excessive alcohol use constitutes an ongoing public health crisis, accounting for ~5% of the global disease burden [3]. Alcohol dependence affects 2.6% of people aged 15+ years worldwide with much higher prevalence rates in many developed countries and causes more harm than illicit drugs [4, 5]. Very heavy drinking (>100 or 60 g/day for males or females, respectively), which for example involves ~0.8% of the population aged 15-65 years in Europe, leads to severe health consequences and dramatically reduced life expectancy [6].

Alcohol dependence (often equated with severe alcohol use disorder, AUD) is characterized by a systematic bias towards choosing alcohol over healthier alternatives, and individuals continue to use alcohol despite adverse consequences, displaying signs of “compulsivity”. This evident resistance to change a dysfunctional behavior demands deeper understanding beyond simply attributing it to individual choice [7]. The question arises: is AUD simply a bad habit? To explore this, we delve into the origin of the term “habit” and its definitions in experimental psychology. According to the recent web edition of Merriam & Webster’s Dictionary [8], the most common use of the term *habit* refers to “a settled tendency or usual manner of behavior” originating from Latin—*Habitus*, but it is also described as “an acquired mode of behavior that has become nearly or completely involuntary”. The latter aspect is also part of how experimental psychology defines habits as learned associations between a stimulus, context or internal state and behavioral responses that become nearly or completely involuntary, independent of the outcome. In contrast, ‘*goal-directed’* behavior is motivated by consequences and requires knowledge of the specific response outcomes. This habit-goal construct is operationalized by testing an operant conditioned response after devaluation of the reward, with the assumption that under habitual control, the response remains unaffected, while under goal-directed control, the subject reduces responding (see Box 1). The traditional dichotomous perspective of the original stimulus-response theory, wherein habitual and goal-directed control are viewed as mutually exclusive, is subject to debate. Contemporary interpretations suggest more nuanced and graded interactions, as elucidated in a recent comprehensive primer on habit theory [9]. Regardless of the deterministic nature of a habit-goal dichotomy, the dominance of excessive or dysfunctional habits has become a common explanation for the transition into compulsivity in drug addiction. Accordingly, prominent theories posit that habit formation indicate diminished control over drug seeking and taking, contrasted with goal-directed responding as a sign of behavioral control [10–14]. Opposing the habit theory of addiction, Hogarth [15] placed excessive goal-directed behavior at the core of addiction development, but this explanation seems equally deterministic as the behavioral automaticity construct.

**Fig. 1:**
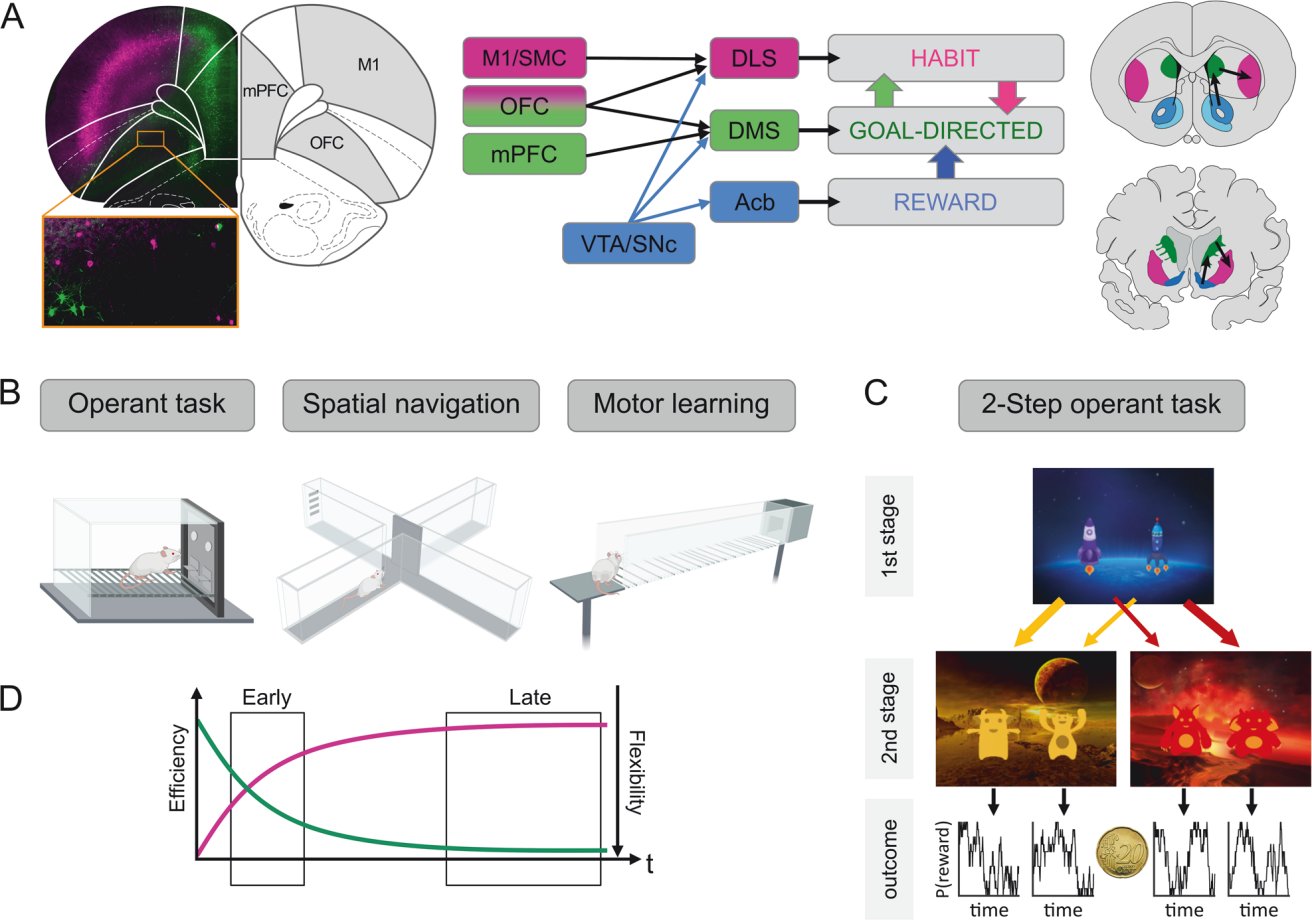
Neurocircuitry and experimental paradigms of striatal learning. **A** *Left:* Corticostriatal projections originate from distinct non-overlapping populations of neurons. Cortical neurons were retrogradely labeled by injection of viral tracers (ssAAV-retro/2-hSyn1-mCherry-WPRE-hGHp and ssAAV-retro/2-hSyn1-EGFP-WPRE-hGHp) within the posterior dorsomedial DMS and anterior DLS. Fluorescence-labeled neurons of the mPFC project to DMS (green), while M1 neurons project to DLS (purple). In the OFC distinct populations are found that project either to DMS or DLS. Scale bar: 1 mm. *Middle:* Simplified representation of the prefrontocortical input to the dorsal striatum. Neurons from M1 and SMC project to the DLS (purple), mPFC neurons to the DMS (green), and OFC neurons project to both regions (purple and green). Striatal dopaminergic input from the VTA and SNc are shown in blue. *Right:* Coronal sections of the rodent and human brain showing the main striatal regions with black arrows representing ventromedial to dorsolateral information transfer. The dorsal part of the striatum can be subdivided into the DMS (rodents) and caudate nucleus (humans) (green) and the DLS (rodents) and putamen (humans) (purple). Functional aspects of this circuitry are described in Box 2. **B** Typical instrumental (operant box), spatial navigation (T-maze), and skilled walking (horizontal ladder) tasks that are used to assess biases of goal-directed or automatic response tendencies in rodents. **C** Human sequential decision-making task (2-step task) to assess model-based and model-free learning (see Box 1). In each trial, participants perform two sequential decisions at two stages in order to obtain probabilistic monetary rewards. In this version [118], participants start from planet Earth (1st stage) and choose between one out of two rockets, in order to land on one out of two planets (2nd stage), each inhabited by two different aliens. Importantly, the transition from 1st stage choice to the 2nd stage underlies a probabilistic structure: while one rocket flies commonly (70% probability) to the yellow planet and only rarely to the red one (30% probability), the inverse structure is true for the other rocket. In the 2nd stage, participants chose between one out of two aliens in order to obtain a reward. The reward probabilities associated with each 2nd stage alien vary slowly across trials according to Gaussian random walks in order to foster continuous learning across the task. That way the 2-step task allows to dissociate model-based from model-free behavior: While pure model-free control simply increases the choice probability of actions rewarded in previous trials, model-based control additionally considers if rewards followed a common or a rare 2^nd^ stage transition, i.e., takes into account the underlying task structure. **D** Schematic learning curve of a behavior. Early into training the behavior may be less accurate or efficient (purple) but will gradually improve. At later time points, performance has stabilized but is less flexible (green) and resistant to interference. Behavioral automaticity such as degree of goal-directed responding can be assessed in early or late phase. Neuroanatomical abbreviations were used according to the rat brain atlas [119]: DLS dorsolateral striatum, DMS dorsomedial striatum, mPFC medial prefrontal cortex, Acb nucleus accumbens, OFC orbitofrontal cortex, M1 primary motor cortex, SMC sensorimotor cortex, SNc substantia nigra pars compacta, VTA ventral tegmental area.

**Fig. 2:**
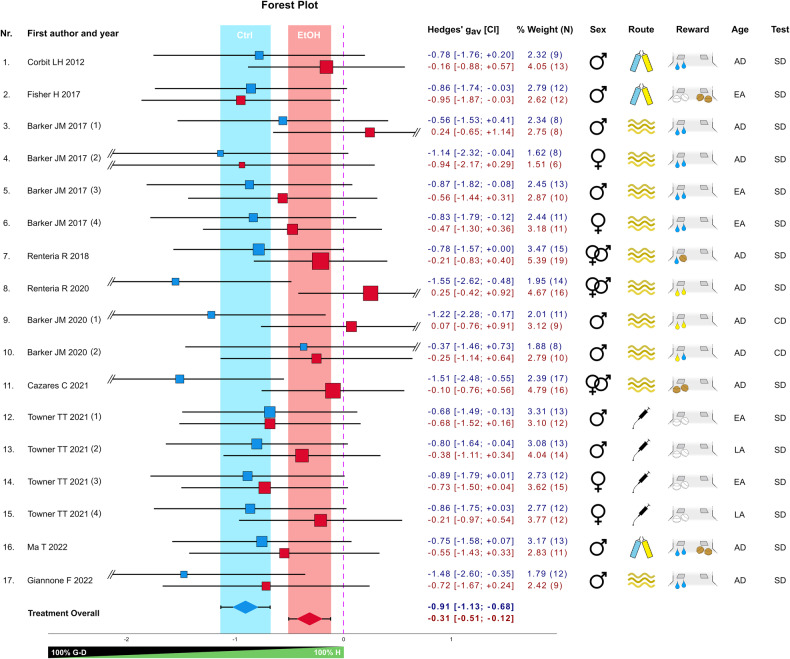
Meta-analysis on the effect of chronic alcohol pre-exposure on responding in reward devaluation tests in rodents. The forest plot shows the standardized effect size (Hedges’ g_av_) representing the difference in responding between reward-devalued and non-devalued conditions of the alcohol and control groups (17 comparisons) from 10 published studies. The experiments included testing different operant schedules (experiments Nr. 3–7), rewards (exp. Nr. 10), reward-lever contingencies (exp. Nr. 16), or time points (exp. Nr. 17). Blue and red squares represent the control and alcohol conditions, respectively, with their position relative to the x-axis indicating the effect size and their area representing their percent weight within the meta-analysis, based on the variance of the effect size. Horizontal lines indicate the confidence intervals (CI), with the values given in the adjacent table. Colored vertical bars represent the CI of the overall effect of control (blue) and alcohol (red) conditions, respectively, also displayed at the base of the plot. The vertical dashed line represents the zero effect, i.e., no devaluation or full habitual behavior. Study variables are shown to the right and include sex and age (early adolescent—EA, late adolescent—LA, adult—AD), route of alcohol administration (oral intake, intra-peritoneal injection, CIE vapor, shown by symbols), and type of reward (white pellets for sucrose pellets, brown pellets for food pellets, blue drops for sweet solution, yellow drops for alcohol solution, shown in symbols), and test condition (satiety devaluation—SD, contingency degradation—CD). Reward symbols beneath the left lever indicate that a single reward was tested (no choice), while those beneath both levers signify that two rewards were tested simultaneously (choice). In exps. Nr. 7 and 10, two rewards were tested separately (no choice).

In this review, we present a contemporary definition of habits and related constructs, along with their experimental operationalization in animals and humans (Box 1). We also explain the main neurobiological concepts related to habitual and goal-directed responding (Box 2, Fig. 1). Against this backdrop, we provide a literature review on animal and human experiments that specifically examined habit and goal-directed behavior in the context of alcohol use and AUD. These studies are compiled in Tables 1 and 2, with a meta-analysis of the animal studies provided in Fig. 2. Most studies assume a competition between goal-directed and habitual control systems, and we conclude that both mechanisms are integral parts of a complex decision-making process. When this process is strongly biased towards automatic responding, it can contribute to the development and maintenance of AUD .

Box 1 Definition and assessment of habits A *Habit* is a rapidly activated specific response in a specific context, which has been repeatedly performed previously, it is in itself inflexible and shows some resistance to change (in context, outcome, motivation). The term “habits” is used on different levels of description, ranging from self-report to experimentally controlled operationalizations. In established operationalization mainly in animal studies, habitual responding is characterized by continued responding despite devaluation of the outcome (probed with devaluation tests) and by an insensitivity towards the causal relation between the response and the outcome (probed with contingency degradation). However, it has been shown that it is difficult to induce habits in human laboratory tasks and that animal paradigms cannot easily be translated to humans. Therefore, computational approaches allow characterizing the degree to which participants flexibly use the causal structure of the environment in goal-directed control or rigidly respond towards certain environmental stimuli in habitual control. A specific response lies on a continuum between habitual and goal-directed control and cannot be easily classified as belonging to one of two dichotomous systems.

*Goal-directed behavior*, which is often contrasted to habits, is performed based on knowledge of the specific outcome and its current motivational value. Thus, the subject distinctly learns the consequences of its action (also termed action-outcome learning, A-O), and thus uses knowledge about the outcome when choosing the action.

*Compulsivity* also refers to automatic behavioral responses but needs to be distinguished from habitual behavior. Compulsivity refers to a resistance to aversive consequences and is modeled in animals as maintaining a certain behavior despite aversive consequences such as electric foot shocks or taste aversiveness (e.g., by the bitter substance quinine). In addiction research, compulsive responding is conceptualized as a severe form of loss of control over behavior, and commonly a transition from habitual to compulsive responding is posited as the disorder progresses [12, 13]. Given the different constructs and assessment methods, it is unclear how a continuum or transition from habitual to compulsive responding can be demonstrated. Indeed, robust correlations between measures of habitual and compulsive responding have not been found [47, 48] and there are some differences in the neurobiology underlying both processes [120] (see also Box 2).

For *assessment of habits* and to distinguish it from goal-directed responses, Dickinson et al. [121] introduced the response to *outcome devaluation* as a discriminative criterion. Thus, if an outcome such as food is devalued, often by satiety or conditioned poisoning in a separate session without the previously reinforced behavior, goal-directed responding will cease while habit-controlled behavior will continue in a test session. Similarly, if the connection between response and outcome is weakened (*contingency degradation)*, for example by suddenly delivering the food independently of a response at the lever, a continued responding would be interpreted as habitual responding. These methods have been shown to work well in animals, especially with food rewards. As expected, following extended training in an operant lever-pressing task, the effect of outcome devaluation is diminished, indicating a heightened manifestation of habitual responding.

*Training schedules* can influence the preferred mode of responding indicating that simply amassing the number of repetitions is not the best training method to induce behavioral automaticity. Thus, habitual responding occurs more often after schedules of reinforcement in which the first response after some variable time (e.g., random interval of 30 seconds, RI-30 schedule) compared to schedules where the rate or number of responses is the relevant factor (e.g., random rate of 10 responses, RR-10), whereby the latter schedule is rather resistant to habit formation [122]. This has become a common way to experimentally induce biases towards one or the other type of behavior and to compare them directly in devaluation tests. Although it is clear from slot machine gambling that uncertainty between action and outcome is a strong driver of habitual behavior, the factors responsible for the different outcomes after RI and RR schedules are not well understood. Such difference may be explained by the action-outcome contingency [121], temporal uncertainty of reward availability [123, 124], or schedule-induced stress [125, 126].

*Skills* describe a learned ability that involves improved performance acquired after extensive training. In contrast to habits, the performance of a skill requires conscious effort to initiate and improve and may therefore be rather goal-directed, the goal being the nearly perfect execution of a more or less complex motor program, for example, riding a bicycle or playing an instrument. However, changing acquired skills, such as switching between automatic and manual transmission cars, can also lead to difficulties. In addition, skills and habits share a common striatal neurocircuitry (see Box 2), but skills emphasize on ‘how the behavior is performed’ whereas habits refer to ‘which stimuli elicit the behavior’ [9, 127]. Skill learning in rodents can be assessed by a variety of tests, for example the skilled walking task [128, 129] (Fig. 1).

*Translation to humans* is well established for the above-mentioned principles of outcome devaluation [57, 59, 60, 130, 131] and contingency degradation [132–134]. However, habit induction in humans has proven difficult: in a large study using various outcome devaluation procedures, de Wit et al. [135]. failed to replicate previous reports that habitual tendencies indeed increase with extended training [57], a negative finding that has been recently reported also in animals [124, 125].

Another approach is based on reinforcement learning theories, distinguishing between habitual and goal-directed behavior as *model-free and model-based control*, respectively [64, 65]. According to this idea, humans either possess a cognitive map, understanding the task’s rules and structure, or simply repeat previously rewarded actions without representing the underlying state transitions. This is exemplified in the 2-step task, where participants make choices in an initial stage and then again in a second stage, leading to potential rewards [136]; Fig. 1. Computational modeling of behavioral responses yields parameters describing model-based and model-free behavior, such as balance parameters, rates of first and second-stage options, and perseverative and prediction errors.

*Self-reports:* Individuals frequently characterize their addictive behavior as a “habit,” yet the precise meaning behind this term often remains ambiguous. To refine this self-reflection, instruments such as the Creature of Habit Scale (COHS) can be utilized [137]. Research on cocaine use disorder has demonstrated a slight but significant increase in automaticity over time, as measured by the COHS, lending support to the validity of this instrument [106]. However, the extent to which the COHS aligns with behavioral assessments of the habit construct, such as reward devaluation tests, is unclear. Notably, the application of the COHS to alcohol use disorder (AUD) remains unexplored.

Box 2 Striatal mechanisms of habit formation The main neurobiological concepts related to habitual and goal-directed responding revolve around the basal ganglia circuitry and propose a shift from dorsomedial (DMS) to dorsolateral (DLS) striatal involvement during the formation and execution of habitual control.

*The cortico-basal ganglia circuitry* is a complex neural network that regulates the affective and motor components of behavior. At its center is the striatum, which receives inputs from various brain regions, including dopaminergic inputs from the ventral tegmental area (VTA) and substantia nigra pars compacta (SNc), as well as glutamatergic inputs from the cortex, hippocampus, and amygdala. The striatum consists mainly of GABAergic medium spiny neurons (MSNs), which play a crucial role in modulating behavior through interactions with these inputs [138].

The striatum can be divided into two parts: the dorsal striatum and the ventral striatum. The ventral striatum includes the nucleus accumbens (Acb) and receives inputs from the VTA and prefrontal association cortices, specifically the medial prefrontal and orbitofrontal areas (mPFC, OFC). In contrast, the dorsal striatum receives inputs from the SNc and primarily from motor cortical regions (primary motor and sensorimotor cortex) but also from association cortices.

Traditionally, dopaminergic projections from the VTA to the ventral striatum are associated with reward processing and reward prediction error, while those from the SNc to the dorsal striatum are implicated in habit formation and motor skill learning, but there is strong overlap between these projections and processes [127, 139]. The output stations of the basal ganglia are the substantia nigra pars reticulata (SNr) and parts of the VTA, which send GABAergic projections to the thalamus. From there excitatory projections regulate activity in cortical fields inputs, completing a feedback loop [138].

Structurally, the striatal circuitry features two important projection patterns generated by the MSNs. One population forms the direct pathway (dMSNs), sending monosynaptic projections to the SNr. The other population forms the indirect pathway (iMSNs), projecting indirectly to basal ganglia output nuclei via the external globus pallidus (GP), the subthalamic nucleus, and other stations. dMSNs and iMSNs are further distinguished by further characterized by expressing dopamine receptors of the D1 or D2 subtype, respectively. Direct and indirect pathways are involved in fine-tuning behavior by providing “drive” or “brake” signals to the thalamus and cortical fields [140, 141]. However, recent research suggests that both dMSNs and iMSNs are concurrently activated during action initiation, with dMSNs exhibiting shorter latency [13].

The striatal circuitry also comprises overlapping spiraling striatal-midbrain-striatal loops organized topologically from ventromedial to dorsolateral regions. This organization is observed not only within the striatal regions but also in the corresponding midbrain, thalamic, and cortical inputs and outputs [142]. This topological organization is exemplarily shown in Fig. 1, with selective projections from mPFC to DMS and from motor cortex to DLS. Intriguingly, the OFC seems to play an intermediary role with distinct populations projecting to either DMS or DLS. These anatomical features enable information transfer from ventromedial to dorsolateral structures during learning of various tasks, such as operant responding, maze navigation, or motor skill learning [9].

*Role of DMS and DLS in the development of behavioral automaticity:* An exemplary demonstration of the medial-to-lateral transition in information processing within the dorsal striatum was observed in mice learning a simple motor task [38]. During the early acquisition phase of learning to stay on a rotating rod, activity was dominated by D1-expressing MSNs located in the DMS. As task performance became consolidated, D2-MSNs of the DLS showed increased activity. This shift was associated with cell type-specific changes in the excitability of MSNs and alterations in regional D1 and D2 expression [38, 143]. Similar shifts in activity patterns were observed in instrumental learning. Inactivation of the DMS led to an accelerated emergence of habitual responding, while lesions of the DLS preserved goal-directed responding even after extended training periods [40, 144]. Mice over-trained in an easy navigation task showed distinct activity patterns in the DLS. Neurons exhibited high activity at the start of the T-maze and at the end when approaching the reward, while during the middle phase of the task, as the mice were crossing the runway, DLS neurons were mostly silent [13]. This pattern of “task-bracketing” activity of the MSNs is coordinated by fast-spiking interneurons and was not observed in the DMS. Instead, DMS neurons fired consistently throughout the performance of a new routine and became disengaged with over-training around the time when task-bracketing in the DLS emerged [38, 100]. These findings suggest that both DMS and DLS regions are involved in parallel processing during initial task learning and when behavioral automaticity is setting in [9, 145]. The shift from DMS to DLS involvement in information processing appears to be critical for the development of habitual control over behavior, highlighting the dynamic nature of the cortico-basal ganglia circuitry in regulating goal-directed versus habitual responses.

*Effects of cortical inputs:* Efforts to understand the distinct roles of cortical inputs in regulating goal-directed and habitual behaviors have primarily focused on mPFC and the OFC. It is commonly believed that prefrontal hypoactivity facilitates habitual responding while activating striatal projections from the OFC and mPFC can counteract this effect [9, 14]. But the picture is more complex. For instance, contrasting effects in the balance between goal-directed and habitual behaviors have been proposed for two subregions of the mPFC: the infralimbic (IL) and prelimbic (PrL) areas. According to some studies, the IL may support habitual behavior, while the PrL promotes goal-directed behavior [146, 147]. Nevertheless, this model appears difficult to reconcile with a similar construct that has emerged in the drug and fear extinction field. This other model suggests that “Go” signals emanate from the PrL, while “No-Go” signals originate from the IL [118, 148]. Combining these findings, one would expect the IL to promote both extinction learning in drug seeking and increased responding in reward devaluation paradigms, and the opposite for the PrL.

*Task-specific neuronal ensembles* may offer a resolution to the discrepancies discussed above. This concept, originally proposed by Hebb [119] to explain the encoding of memories, has now been further developed to elucidate the reactivity to cue-reward associations. According to this concept, specific functions or tasks are encoded by discrete populations of neurons with distinct cell type identity, connectivity, and temporal coactivation patterns [149–151]. Local ensembles are topographically dispersed across the circuitry and form rapidly shifting meta-ensemble networks that support efficient and flexible on-demand decision-making [96]. The observation of response-specific dynamic network configurations, encompassing sparsely distributed neuronal populations from various brain regions, adds an additional layer of complexity to the encoding of different types of response probabilities. This complexity argues against models of dichotomous control, not only between DMS and DLS but also among various prefrontal inputs.

*Brain mechanisms of compulsivity:* Neural substrates of persistent responding despite negative consequences have been identified in circuits that are involved in emotional, social, and stress processing, such as insular cortex, amygdala, and the midbrain origins of the serotonergic and noradrenergic system [50, 152, 153]. Activation of these circuits influences the striatal circuitry. Additionally, prefrontal hypoactivity is widely believed to facilitate compulsive responding, while activating mPFC or OFC projections to the striatum can counteract it [12, 154]. An alternative role of the OFC in compulsivity has been proposed by Pascoli et al. [155]. In a new mouse model of addiction based on excessive optogenetically mediated self-stimulation of the VTA, the authors demonstrated that potentiation of synapses from the lateral OFC onto the dorsal striatum was associated with compulsive (punishment-resistant) responding. They concluded that an overactive OFC-dorsal striatal pathway could lead to an overestimation of the value of drug experience relative to punishment, biasing instrumental behavior towards drug-taking. Thus, the OFC appears to play a critical role in either facilitating or counteracting automaticity, likely depending on interactions of these projections with further inputs from motor or associative cortical areas.

Thus, distinct neural mechanisms have been identified that facilitate compulsive responding for rewards. Some of these mechanisms may overlap with the substrates underlying habitual response biases, but specific evidence for a mechanistic continuum from habitual-biased to persistent compulsive responding, as proposed by some addiction theories [12, 13] has not been presented so far.

*Human brain data:* In humans, examining experimental habit formation has been shown to be challenging e.g., de Wit et al. [135] and Tricomi et al. [57] were the first to demonstrate habitual behavior after extensive training in a free operant learning task, showing increasing activity in the dorsolateral posterior putamen as a neural correlate of habit formation. Interestingly, magnetic resonance spectroscopy studies revealed reduced glutamate turnover in the putamen of patients with cocaine use disorder, suggesting dysregulation of glutamatergic transmission in this region caused by chronic cocaine use [106]. This neurochemical deficit was related to increased habitual responding in a contingency degradation test. Additionally, investigations employing the 2-step task and its computational framework of reinforcement learning [136]; Box 1 and Fig. 1, have revealed specific roles for the ventral striatum during model-free learning and the ventral mPFC during model-based behavior [for meta-analysis], see [156].

Overall, the neurobiological data from both animal and human paradigms show some differences in processing between DMS and DLS during the transition from goal-directed to habitual control of responding. However, there is no consistent support for the common notion that the DLS is universally promoting automaticity, while the DMS may oppose it and instead promote goal-directed behavior. More likely are parallel information processing modes that allow rapid reorganization of behavioral strategies upon demand [9].

## Animal studies

Laboratory animals easily acquire lever-pressing behavior for rewards (instrumental learning), typically food but also alcohol and other addictive drugs used by humans. After multiple self-administration sessions, animals may increase alcohol intake, which is likely due to the fact that alcohol is initially aversive to most rodents. Animals will reach a stable level of alcohol-self-administration, though this basal level of alcohol consumption is deemed not to reflect any addiction-like behavioral feature [16]. Also, such initial escalation is absent when food or other non-drug rewards drive motivation. However, animals can substantially escalate their alcohol intake in response to specific experimental manipulations (e.g., scheduled access, distinct cues or contexts, stress), which prompts inquiries about whether this increase reflects a loss of control over seeking and taking behavior, and to what extent habitual responding contributes to this phenomenon.

Rodent studies have mainly explored two key facets of alcohol’s impact on habitual responding after reward devaluation (for assessment of habitual or goal-directed responding see Box 1). One line of research investigates whether alcohol reinforcement exhibits more robust habit-forming properties than the consumption of food or other natural rewards. Another critical focus revolves around the consequences of prolonged or excessive alcohol exposure on habitual control and its potential generalization to other rewarding non-drug stimuli.

In a pioneering study, Dickinson and colleagues trained rats to press different levers for reinforcement by either alcohol solution or food pellets [17]. Results showed resistance to outcome devaluation (by lithium chloride poisoning) in the alcohol but not the food condition. In a similar study examining self-administration of sweetened alcohol and sugar solution in rats, findings showed after short training persistent responding following reward devaluation only for the alcohol group at, while after extended self-administration training both groups showed persistent responding in the devaluation test [18].

In a seminal study, Corbit et al. [19] examined the effects of training duration on rats self-administering alcohol. After 4 weeks of training, alcohol self-administering rats showed reduced sensitivity to satiety devaluation, while rats self-administering sucrose remained sensitive even after 8 weeks of training. However, sensitivity to satiety devaluation was not maintained in rats with additional non-contingent access to alcohol in their home cage. Moderate levels of regular alcohol consumption, below 0.5 g/kg/day, correlated with the degree of habitual responding, even without noticeable intoxication. Interestingly, inactivation of the dorsomedial striatum (DMS) led to faster development of habitual responding, while animals receiving lesions of the dorsolateral striatum (DLS) after 8 weeks of training restored goal-directed responding (for a brief primer on neurobiological mechanisms see Box 2). Follow-up experiments demonstrated that habitual alcohol self-administration was driven by dopamine D2 receptor and ionotropic (AMPA) glutamate receptor activation within the DLS [20]. Moreover, alcohol also induced increased AMPA receptor activity and dendritic branching in the DMS, specifically in D1-expressing medium spiny neurons (D1-MSNs) [21]. Simultaneous recordings from DMS and DLS neurons in rats self-administering alcohol by Fanelli et al. [22] demonstrated concomitant but specialized phasic firing patterns, where DMS neurons fired mostly time-locked to reinforcement or reward-predicting cues, while DLS activity was associated with lever-pressing, with minor differences under different schedules of reinforcement. Thus, on a structural or functional level there is little support for two opposing systems supporting either habitual and goal-directed control. Instead, distinct populations of neurons in the DMS and DLS may serve specific aspects of a behavioral control, and these populations respond differently to chronic alcohol exposure.

Collectively, these studies provide evidence supporting alcohol’s greater and faster potential to induce habit-forming effects compared to other non-drug rewards. It is worth noting that, except for Dickinson et al. [17], most studies used test paradigms with only one instrumental response. Such a limited decision space may not challenge cognitive resources to an extent that favors reliance on an automatic control system. In a notable experiment by Ostlund et al. [23], rats presented with two simultaneous food rewards exhibited reduced goal-directed control over instrumental responding in a context previously associated with alcohol (i.p. injections), but not with saline cues, highlighting the disruptive influence of alcohol-paired cues on decision-making and goal-directed actions.

Also to mention, Shillinglaw et al. [24] failed to find evidence of habitual responding for alcohol or sucrose. The experiment assessed satiety devaluation and contingency degradation (the latter only at the end of the experiment). Besides, rats trained on low-caloric alternatives, such as 1.5% sucrose or non-sweet 10 mM monosodium glutamate, displayed insensitivity to reward devaluation by satiety but not by contingency degradation (reward delivery independent of the lever response). This dissociation between the two main reward devaluation methods suggests potential differences in the underlying neural mechanisms, although the study did not explore this aspect further.

Recent studies have focused on whether alcohol’s long-lasting (i.e., non-pharmacological) effects may affect decision-making systems underlying goal-directed actions, potentially leading to habitual tendencies. Pre-exposure to alcohol can occur through voluntary home cage access, scheduled operant self-administration or passive exposure via various administration routes. Among the latter, chronic intermittent alcohol vapor exposure (CIE) has become a popular rodent model of AUD, ensuring clinically relevant high blood alcohol levels (>1.5 g/l) and safely administered over weeks [25–27].

The Gremel lab conducted three studies using the CIE paradigm to investigate alcohol dependence’s effects on orbitofrontal cortex (OFC) function in goal-directed behavior. Mice with CIE treatment showed insensitivity to satiety devaluation, associated with alterations in OFC top-down control on striatal circuits. CIE reduced OFC excitability, and artificially increasing activity of OFC projection neurons during protracted abstinence restored sensitivity to outcome devaluation. In vivo extracellular recordings during the operant task revealed a long-lasting disruption in OFC function due to CIE, leading to enhanced activity associated with actions (lever response) but diminished activity during outcome-related information (reward collection) [28–30]. Thus, chronic alcohol exposure alters OFC activity critical for decision-making processes biasing for habitual responding, but the specific contributions of OFC need further study.

Barker et al. [31] found that mice with CIE treatment prior to operant training displayed habitual tendencies for alcohol self-administration, but not for sucrose. Pre-exposure to chronic alcohol appeared to impair goal-directed alcohol-seeking more than sucrose-seeking behavior. However, another study using a high-alcohol diet for about 3 weeks found no effect on responding in a devaluation test [32]. Similarly, two other studies with chronic voluntary alcohol consumption prior to instrumental training and testing under conditions of simultaneously available devalued and non-devalued options reported no effects on outcome devaluation [33, 34]. The lack of effect on outcome devaluation may be due to lower alcohol exposure compared to the CIE paradigm. In the study by Ma et al. [34], alcohol-drinking rats displayed insensitivity to outcome devaluation when cognitive load was increased by contingency reversal (rewards were switched relative to the levers), suggesting that in rats with a chronic drinking, history engagement of habitual response strategies may occur with higher cognitive demands. This behavioral shift was associated with compromised function of cholinergic interneurons in the DMS, which regulate the activity of D1 and D2 receptors-containing MSNs, influencing behavioral flexibility. Optogenetic enhancement of thalamic input to these interneurons reduced the bias towards habitual responding in chronically alcohol-drinking rats.

Three studies explored age and sex influences on alcohol-induced habitual tendencies. Barker et al. [35] found sex differences after CIE. Operant responding for sucrose after CIE was less sensitive to satiety devaluation in adults compared to younger males, and only adolescent female rats showed habitual tendencies, indicating reduced behavioral control in younger females. On the other hand, chronic high alcohol exposure during late adolescence increased habitual responding in adulthood regardless of sex [36]. Even without a history of alcohol dependence, developmental differences in habitual tendencies towards alcohol reward were observed, with higher susceptibility in adults compared to adolescent rats [37]. The results suggest that susceptibility to alcohol-induced habit formation increases during the transition from adolescence to adulthood, particularly in male rats. However, more research is needed to better understand the effects of sex and age.

The question arises whether similar striatal learning processes, involving information transfer from medial to lateral structures (Box 2, [9, 38]), are mediated by the same striatal cell population. We conducted experiments with rats trained on a T-maze and an instrumental task and found that prior CIE treatment led to increased automatic responding in both tasks [39]. In addition to reduced sensitivity to reward devaluation, CIE-rats made more errors in a well-learned spatial navigation task, indicating the impact of chronic alcohol dependence on various aspects of action control beyond instrumental learning. These behavioral changes were strongly dependent on DMS function. Chemogenetic inhibition of this region increased habitual bias in normal rats, aligning with previous findings that suggest a key role of the DMS in both tasks [40, 41]. These experiments suggest overlapping cell populations controlling different behaviors beyond instrumental performance, implying that alcohol’s detrimental effects on these cells may not only affect reward-seeking but also other behaviors relying on striatal learning.

Certain pharmacologically targetable mechanisms have been investigated to understand their role in habitual biases. Notably, increased endocannabinoid signaling via CB1 receptors in the DLS appears to be crucial for habitual tendencies. Studies have shown that inhibitors of endocannabinoid synthesis or transport, as well as CB1 blockade, reduced responding for alcohol cues after contingency degradation or lithium devaluation, while CB1 agonists enhanced habitual responding [42]. The higher abundance of CB1 receptors in the DLS compared to the DMS allows for target specificity in systemic pharmacological approaches. Additionally, chronic alcohol exposure has been associated with neuroadaptations, including increased CB1 signaling, enhancing DLS control in learning [43]. These findings suggest the possibility of pharmacological interventions targeting habitual biases.

Moreover, injection of rapamycin, a specific mTORC1 inhibitor, into the OFC of chronically drinking rats reduced habitual responding for alcohol [44]. This effect is attributed to mTORC1’s role in local dendritic translation of synaptic proteins. Notably, mTORC1 is activated via phosphorylation by GluN2B, a subunit of the NMDA-type glutamate receptor complex, which is upregulated after prolonged alcohol exposure in the corticostriatal circuitry [45, 46]. Thus, mTORC1 signaling appears to be a critical mediator of alcohol-induced synaptic plasticity, and inhibition of this pathway may offer the potential to reverse these neuroadaptations and improve control over alcohol intake.

**Table 1:** Animal studies investigating the effects of alcohol on goal-directed vs. habitual control.

Study	Species (Sex) Age	Alcohol pre-exposure (length)	Reward(s)	Training schedule/Devaluation method	Main results
Dickinson et al. [17]	Rats (♂) AD	-	EtOH & food pellets (CHOICE)	RI 30/LiCL	EtOH seeking is habitual; Food seeking is goal-directed
Ripley et al. [32]	Rats (♂) n/r	liquit diet (≥24 d with or without intermittent withdrawal)	SUC	RI 30/LiCl (with feedback)	EtOH diet does not affect habitual seeking for SUC. Context of LiCl devaluation does: devaluation in the operant boxes induces goal-directed seeking, contrary to devaluation in the home cages, after possibility of re-experiencing the reward is given.
Ostlund et al. [23]	Rats (n/r) AD	i.p. (14 d: 7 d per context)	Grain & SUC pellets (CHOICE)	RR 10/SD	Seeking for a natural reward becomes habitual in an EtOH paired context
Mangieri et. al. [18]	Rats (♂) AD	-	EtOH/SUC & SUC	RI 30/LiCl	Sweetened EtOH, but not SUC, seeking becomes habitual after short training
Corbit et al. [19]*	Rats (♂) AD	2BC (CA 14 d prior training then IA during training)	EtOH & SUC	RR 3/SD	EtOH seeking becomes habitual after four weeks, SUC seeking remains goal-directed after eight. Non-contingent EtOH drinking renders SUC seeking habitual. Neurocircuitry: DMS inactivation induces habitual seeking after short training; DLS inactivation maintains goal-directed seeking after long training.
Fanelli et. al. [22]	Rats (♂) AD	-	EtOH	FR 5 & RI 30/SD & CD	Animals self-administering EtOH show DMS activity spikes in response to reward release and cue presentation, DLS activity spikes are associated with lever-pressing.
Shillinglaw et al. [24]	Rats (♂) AD	-	EtOH; SUC; MSG	FR 5/SD & CD	10%SUC and 10%EtOH seeking remain goal-directed after long training, unlike 1.5% SUC and MSG seeking, when tested via SD. CD test shows goal-directed seeking for all rewards.
Corbit et al. [20]	Rats (♂) AD	2BC (CA 14 d prior training then IA 14 d)	EtOH	RR 3/SD	D2 and AMPA receptors in the DLS mediate habitual EtOH seeking. D2R and AMPAR antagonists (raclopride and NBQX respectively) into the DLS restore goal-directed seeking.
Serlin & Torregrossa [37]	Rats (♂) EA&AD	-	EtOH/SACC	RI 30/CD	Adult animals develop habitual EtOH seeking after short training, adolescents remain goal-directed after prolonged training while consuming more EtOH.
Barker et al. [35]*	Rats (♂&♀) EA & AD	CIE vapor (2 weeks)	SUC	RI 60 & RR 10/SD	Females exposed to EtOH during adolescence show increased habitual seeking in adulthood, not males. Males exposed during adulthood show increased habits, not females
Fisher et al. [33]*	Rats (♂) EA	2BC (IA 6 w)	Precision & SUC pellets (CHOICE)	RI 20/SD	EtOH pre-exposure does not induce habitual seeking for a natural reward in a choice setting
Renteria et al. [28]*	Mice (♂&♀) AD	CIE vapor (4 weeks)	Food pellets & SUC	RI 60 & RR 20/SD	EtOH exposure induces habitual seeking under both RR and RI schedules, controls show habits only under RI schedule. Neurocircuitry: EtOH reduces OFC → DMS excitatory neurons’ activity. Their activation restores goal-directed seeking in EtOH exposed animals.
Houck & Grahame [117]	Mice (♂&♀) AD	i.p. (acute)	flavored water	RI 60/LiCL	Acute EtOH intoxication induces habitual seeking
Morisot et al. [44]	Rats (♂) AD	2BC (7 weeks)	EtOH	RR 3 & RI 30/SD	Inhibition of mTORC1 in the OFC of animals pre exposed to ETOH restores goal-directed EtOH seeking without affecting SUC seeking.
Renteria et al. [29]*	Mice (♂&♀) AD	CIE vapor (4 days)	EtOH	RR 4/SD	EtOH exposure induces habitual EtOH seeking and altered consummatory behavior, with longer drinking bursts and more licks per burst.
Gianessi et al. [42]	Mice (♂) AD	-	EtOH/SACC	RI 60/CD & LiCL	Systemic inhibition of endocannabinoid production or transport or inhibition of CB1R reduces habitual EtOH seeking.
Barker et al. [31]*	Mice (♂) AD	CIE vapor (2 and 4 weeks)	EtOH & SUC	RI 30/CD	EtOH exposure induces habitual seeking for EtOH but not for SUC
Towner & Spear [36]*	Rats (♂&♀) EA&LA	Oral gavage (20 days)	SUC pellets	RI 60/SD	EtOH exposure during late adolescence induces habitual seeking regardless of sex, males and females exposed during early adolescence maintain goal-directed seeking.
Cazares et al. [30]*	Mice (♂&♀) AD	CIE vapor (4 weeks)	Food pellets	ADT/SD	EtOH exposure induces habitual food seeking. Neurocircuitry: EtOH exposed mice show reduced OFC activity during reward-approaching but higher activity during lever-pressing behaviors.
Giuliano et al. [48]	Rats (♂) LA	2BC (12 days prior–10 days during)	EtOH	RI 60–FR 1 (seeking-taking chain)/Extinction of taking behavior	Alcohol-preferring rats pre-exposed to EtOH and that showed strong habitual EtOH seeking were more likely to develop EtOH compulsive seeking later on in training.
Smeets et al. [47]	Rats (♂) AD	2BC (8 weeks prior–every weekend during)	EtOH	RR 3/SD	AUD-like rats, identified based on four phenotypic responses (home cage drinking, habits, motivation, and compulsion) from a pool of animals with prolonged EtOH access, display increased alcohol intake, motivation and compulsion when compared to non-AUD-like rats but same habitual behavior.
Ma et al. [34]*	Rats (♂) AD	EtOH 2BC (8 weeks)	Food pellets & sucrose solution (CHOICE)	RR 20/SD	EtOH exposure does not induce habitual seeking for a natural reward in a choice setting, when lever-reward contingency is reversed habitual seeking appears only in EtOH exposed animals. Neurocircuitry: EtOH exposure impairs CIN-mediated inhibition in D1-MSNs. Optogenetic potentiation of thalamostrital transmission in DMS CINs goal-directed control after lever-reward contingency reversal.
Giannone et al. [39]*	Rats (♂) AD	CIE vapor (7 weeks)	SACC	RI 30/SD	EtOH exposure increases habitual tendencies in both operant conditioning and spatial navigation paradigms. Neurocircuitry: Chemogenetic inhibition of pDMS in EtOH non-exposed animals induces similar increased habitual tendencies in both paradigms.

Studies included in the meta-analysis are marked with an asterisk (*).

♂ and ♀: males and females, *AD* adult (≥52 PND), *EA* early adolescent (25–44 PND), *LA* late adolescent (45–50 PND), *2BC* 2-bottle choice, *i.p*. intraperitoneal, *CIE* chronic intermittent vapor exposure, *SUC* sucrose, *SACC* saccharin, *EtOH* ethanol, *CHOICE* choice present during test, *RI* random interval, *RR* random ratio, *SD* satiety devaluation, *CD* contingency degradation, *LiCl* conditioned taste aversion, *DMS* dorsomedial striatum, *DLS* dorsolateral striatum, *OFC* orbitofrontal cortex.

Two studies examined whether insensitivity to outcome devaluation could be linked to resistance to punishment (compulsivity) and serve as a predictor of addiction progression. In a large cohort of male rats assessed over 60 weeks of alcohol access, an addiction severity score was computed based on various measures related to AUD [47]. This score identified a small group (5 out of 47 rats) displaying higher alcohol intake, increased motivation under a progressive ratio schedule, and reduced sensitivity to quinine adulteration, indicative of compulsivity. Surprisingly, these AUD-like rats did not differ from non-addicted rats in the satiety devaluation test after long-term operant self-administration training. In contrast, Giuliano et al. [48] found that individual differences in habitual control over alcohol seeking predicted the development of compulsive alcohol intake. Rats trained in an operant seeking-taking chain for alcohol self-administration displayed habitual tendencies, with the majority (17 out of 26) becoming resistant to outcome devaluation. Subsequent tests for compulsive behavior, including footshock during seeking and adulterated alcohol drinking, showed that a minority (7 out of 24) exhibited signs of compulsive intake, with six previously identified as habitual responders in the outcome devaluation test. It is worth noting that although the study suggests a connection between habitual seeking and compulsive intake, the majority of rats displaying habitual tendencies (10 out of 17) did not progress into compulsive alcohol intake.

Importantly, both experiments showed that despite long-term alcohol access, only a minority of rats developed addiction-like behavior characterized by resistance to negative consequences. This is consistent with other studies indicating that around 15–30% of outbred rats spontaneously exhibit persistent ethanol intake despite quinine adulteration or foot shock punishment, likely due to distinct genetic factors [49, 50]. Whether these factors influence the development of habitual responding remains uncertain.

## Meta-analysis of rodent studies

We conducted a meta-analysis to address the diverse experimental variations in the aforementioned reports and draw robust conclusions. Therefore, we calculated standard effect sizes for each experiment by normalizing the difference in responding between reward-devalued and non-devalued conditions, irrespective of the specific reward devaluation used (satiety or contingency degradation). The meta-analysis included 10 studies with 17 independent experiments, totaling 404 animals. The 10 studies are marked in Table 1 and discussed in the section above.

### Method

We conducted a PubMed search in December 2022 using the keywords: “(alcohol OR “alcohol addiction” OR “alcohol dependence”) AND (habits OR “habitual behavior”) AND (rats OR mice OR rodents)”. Initially, we screened 202 studies based on their abstracts to exclude non-relevant studies. The remaining papers were then assessed based on their full content. For studies deemed relevant after the full-text screening, we further examined their bibliographies to identify additional pertinent studies. From the refined selection, 10 studies were identified that compared alcohol pre-exposure versus control condition. We measured the effect size of devaluation/contingency degradation for both groups in each experiment from the selected studies, resulting in 25 total comparisons with 203 exposed and 201 non-exposed animals. Means and standard deviations (SD) from pre-test/test conditions were extracted from the graphs using WebPlotDigitizer 4.6. Given that the effect of interest was, for all experiments, derived by comparing a pre-test (non-devalued–non-degraded) to a test (devalued–degraded) condition from the same subjects in a repeated or matched design, we first calculated the Cohen’s d_av_ [51], which is the ideal choice when the correlation coefficient “r” between the dependent measures is not available [52]. This was then converted to Hedges’ g_av_ to correct for positive bias arising from small sample size [51, 53]. In situations where the same animals underwent two different satiety devaluation or contingency degradation tests under different instrumental conditions (e.g., varied schedules or rewards) resulting in two effect size measures from the same set of animals, such effects were averaged into one representative effect size to avoid inflation of the sample size [52, 54], resulting in 17 final ethanol versus control group comparisons. The variance for each individual effect size was calculated assuming r = 0, in order to avoid overestimation of the confidence of the effect size [53]. The variance of the representative averaged effect sizes derived from two dependent effects were calculated assuming r = 1, for the same purpose [53]. The methodology that we used for estimating the variance associated with the effect sizes was intentionally conservative: our primary objective was to rigorously test the robustness of the observed difference between control and ethanol-treated conditions. If an effect is found to be significant under these conditions, it is safe to assume that it would likely become more significant under less conservative (and more realistic) variance assumptions. Finally, we performed a subgroup meta-analysis comparing ethanol-treated/non-treated conditions using SPSS 29 with a random-effect model/REML estimator to account for between-study variability. Publication bias was assessed via Egger’s test, while heterogeneity across experiments was evaluated via I^2^. We additionally tested the robustness of our findings by employing different sensitivity analyses. For detailed explanation of the effect size and variance calculations, and of the sensitivity analyses used, see the Supplementary information.

### Results

Our meta-analysis shows that alcohol treatment significantly affects animal behavior when tested for habitual responding as indicated by the forest plot (Fig. 2) and confirmed via meta-regression analysis (effect of Treatment: t = −3.891; p < 0.001 [CI = −0.903; −0.283]). Specifically, a zero difference between the pre-test and test condition indicates complete habitual behavior, while a decrease after devaluation signifies the degree of goal-directedness. The forest plot revealed a highly significant effect, indicating reduced goal-directed behavior in alcohol-exposed groups compared to controls. Notably, the alcohol-treated animals also significantly differed from zero, showing that chronic alcohol exposure didn’t trigger an all-or-nothing shift to habitual behavior but rather led to a gradual reduction in goal-directed responding. Meta-regression analysis indicated that various experimental factors, such as animal characteristics, alcohol exposure details, training parameters, and reward type, had no significant influence. There was no clear evidence of publication bias (Egger’s test—Ctrl: p = 0.800; EtOH: p = 0.196; Overall: = 0.012; Supplementary Figs. 1, 2), and heterogeneity across experiments was low [55] with I^2^ = 0.07. Finally, we confirmed our meta-analytical results by employing both “leave-one-out” and correlation coefficient sensitivity analyses (see Supplementary Information). Overall, our meta-analysis suggests a dimensional relationship between habitual and goal-directed control, which is compromised by prolonged or chronic alcohol exposure, rather than supporting a clear dichotomy between the two.

## Human studies

We found 9 human studies exploring the balance between goal-directed and habitual choice tendencies either in AUD and high-risk populations, or associations with AUD severity in large community samples (Table 2). Among these studies, seven employed a sequential decision-making task to distinguish between goal-directed (model-based) and model-free learning systems, while two used an outcome devaluation procedure. The focus of these investigations was primarily on instrumental habitual versus goal-directed decision-making related to non-drug rewards and contexts. In other words, they examined generalized habitual response tendencies for newly learned instrumental contingencies within a single session.

**Table 2:** Human studies investigating goal-directed vs. habitual control in AUD and at-risk populations.

Study	Sample	Abstinence duration	Test type & reward	Main results
Sjoerds et al. [56]	31 AUD (18 ♂) vs. 19 controls (12 ♂)	12.3 ± 21.2 days (min. 24 h)	instructed outcome devaluation; [points, transformed to € after the task]	behavior: AUD vs. HCs showed reduced R-O knowledge during outcome devaluation test; ∅ group differences during instrumental learning fMRI: AUD vs. HCs: showed reduced vmPFC and anterior putamten activation during goal-directed learning, and increased posterior putamen activation during habitual learning; ∅ associations with abstinence duration
Sebold et al. [66]	26 AUD (21 ♂) vs. 26 controls (21 ♂)	15.1 ± 10.4 days (min. 2 days)	Two-step task; [Probabilistic monetary reward]	AUD vs. HCs sowed reduced MB control, especially following non-rewards; ∅ group differences in MF learning
Voon et al. [67]	30 AUD (18 ♂) vs. 90 controls (54 ♂)	16.2 ± 16.1 weeks (min. 2 weeks)	Two-step task; [Probabilistic monetary reward]	∅ group differences in MF/MB control; positive association between model-based behavior and abstinence duration
Gillan et al. [72]	Community sample of 1413 participants (590 ♂) with no known diagnosis		Two-step task; [Probabilistic monetary reward]	negative association between MB control and alcohol use (AUDIT score); negative association between transdiagnostic latent factor ‘compulsive behavior and intrusive thought’ and MB control
Sebold et al. [68]	90 AUD (divided at 1-year follow-up in 37 abstainers (30 ♂) vs. 53 relapsers (47 ♂)) vs. 96 controls (80 ♂)	abstainers: 21.4 ± 11.6 days at MRI session relapsers: 22.3 ± 12.4 days at MRI session	Two-step task; [Probabilistic monetary reward]	behavior: ∅ overall group differences in MB control; interaction between MB control and alcohol expectancies predicted group membership (prospective relapsers vs. abstainers vs healthy controls); fMRI: relapsers showed reduced MB RPEs in mPFC compared to abstainers and healthy controls
Doñamayor et al. [69]	31 severe binge drinkers (19 ♂) vs. 35 controls (15 ♂)		Two-step task; [Probabilistic monetary reward]	binge drinkers vs. HCs showed reduced MB control; ∅ association with alcohol use (AUDIT score)
Nebe et al. [70]	188 18-year-old male social drinkers		Two-step task; [Probabilistic monetary reward]	∅ associations of behavioral or neural markers of MF/MB control with alcohol consumption or binge-drinking
Patzelt et al. [74]	community sample of 839 participants (51.25% ♂) with no known diagnosis		Two-step task; [Probabilistic fictive reward]	∅ association between alcohol use (AUDIT score) and MF/MB control
van Timmeren et al. [60]	38 AUD (27 ♂) vs. 22 controls (17 ♂)	7.1 ± 6.6 days (min. 2 weeks)	taste aversion induced outcome devaluation; [snack food]	∅ group differences on behavioral or neural level; ∅ associations with abstinence duration or AUD severity
Chen et al. [71]	146 young male social drinkers at age 18–21 (3-year follow-up sample from Nebe et al. 2018)		Two-step task; [Probabilistic monetary reward]	MB control was negative predictor of binge-drinking trajectories; model-free RPE signals in vmPFC and VS were a positive predictor of alcohol consumption trajectories

*AUD* alcohol use disorder, *HCs* healthy control participants, ♂ males, *MB* model-based, *MF* model-free, *R-O* response-outcome, *(v)mPFC* (ventro)medial prefrontal cortex, *VS* ventral striatum, ∅ not evident.

Currently, there is a lack of human studies investigating contingency degradation sensitivity in AUD or related conditions. Sjoerds and colleagues [56] conducted a study using an outcome devaluation procedure, which included instrumental learning and an outcome devaluation test. The results revealed that abstinent participants with AUD showed impaired action-outcome knowledge compared to healthy controls, indicating a greater reliance on habitual stimulus-response associations rather than goal-directed associations when learning new instrumental contingencies. Interestingly, participants with AUD exhibited increased posterior putamen activity during habitual learning, while control participants showed stronger BOLD-responses in vmPFC and anterior putamen during goal-directed learning. These findings are consistent with animal and human evidence highlighting the distinct roles of vmPFC and anterior putamen in supporting goal-directed behavior, while the posterior putamen plays a key role in habitual behavior (Fig. 1, Box 2) [57, 58]. However, AUD duration did not significantly correlate with behavioral or neural indices of goal-directed or habitual control [56]. It is important to note that the task utilized by Sjoerds et al. [56] lacked the slips-of-action test phase introduced in later task versions (e.g., de Wit et al. [59]), where instrumental responses for devalued vs. non-devalued outcomes are tested in extinction, providing a more direct translation from classical animal paradigms.

An interesting aspect of the study by Sjoerds et al. [56] is the use of two task versions, one with drug-unrelated stimuli (fruits) and the other with pictures of alcohol. Both behavioral and neural results did not differ between the two versions, suggesting that the alcohol binge context did not differentially influence habitual choice tendencies in individuals with AUD. Van Timmeren et al. [60] employed a different variant of the contingency degradation paradigm, incorporating a Pavlovian-to-Instrumental Transfer test [61] along with fMRI to compare abstinent AUD participants with a control group. During instrumental training, participants were trained to respond with left or right button presses, each associated with a different food snack. Subsequently, one of the outcomes was devalued using magnesium sulfate solution to induce a bitter taste and a video displaying waxworm-infested food. Both AUD and control participants showed significant devaluation effects, suggesting intact goal-directed control in AUD. Neuroimaging analysis comparing choices for devalued and non-devalued outcomes revealed no group differences or main task effects. It is essential to note that the two outcome devaluation studies differ considerably in their approach. Sjoerds et al. [56] assessed explicit response-outcome (R-O) knowledge after instructed devaluation, while van Timmeren et al. [60] investigated free instrumental responding after taste aversion-induced devaluation.

Indirect support for a shift from ventral to dorsal striatal activity was shown by Vollstädt‐Klein et al. [62], with reduced neural alcohol cue reactivity in heavy drinkers compared to social drinkers in the ventral striatum. In a follow-up study by Hornoiu and colleagues [63], self-reported automated alcohol craving and habitual alcohol consumption correlated with increased activation in dorsal striatal, pallidal, and prefrontal regions during the alcohol cue-reactivity task.

Besides the translational attempts from animal models, another line of human habit research has formalized habitual and goal-directed processes within a reinforcement learning framework in terms of model-free and model-based control, respectively (see Box 1, Fig. 1) [64, 65]. Sebold et al. [66] compared abstinent AUD participants and controls on performance in the 2-step task, finding reduced model-based, but unchanged model-free control in the AUD group. Model-based control specifically impaired in the non-reward condition, was attributable to cognitive speed differences between groups, highlighting the need to consider potential confounding factors. Further studies by Voon et al. [67], and Sebold et al. [68], did not find direct evidence of reduced model-based control in AUD participants. Nevertheless, model-based control predicted relapse status during a follow-up assessment, and prospective relapsers showed attenuated neural signatures of model-based control in the mPFC compared to controls and abstainers [68]. Additionally, the balance parameter ω scaled positively with abstinence duration in AUD participants [67]. Overall, these findings suggest that reduced model-based control may mediate relapse risk in AUD, but this impairment can recover with prolonged abstinence.

Doñamayor et al. [69] studied young severe binge-drinkers and controls, finding reduced model-based control in binge-drinking participants. Additionally, binge drinkers showed lower learning rates for first-stage options and increased perseverative errors in the 2-step task. However, Nebe et al. [70]., using a less strict criterion for binge-drinking in a community sample of 188 young male social drinkers (i.e., at least one-lifetime binge-drinking episode), found no differences in behavioral model-based vs. model-free control or associated neural reward-prediction error signals. They also found no correlations between these control measures and average alcohol consumption or age at drinking onset. A 3-year follow-up of the same cohort revealed that lower behavioral model-based control was associated with the development of binge-drinking over time, while increased model-free reward prediction error signals in ventral striatum and vmPFC were linked to increased alcohol consumption [71]. These findings complement Sebold et al. [68]. by suggesting the predictive power of the model-based and model-free learning balance for treatment outcomes and drinking trajectories.

Two online studies explored symptom dimensions across diagnostic categories related to goal-directed control. Gillan et al. [72]. found in a population sample of nearly 2000 participants a weak but significant negative association between model-based control and alcohol use severity assessed by AUDIT [73], specifically related to compulsive behavior and intrusive thoughts. Another online study found alcohol use to be unrelated to model-based control in a non-patient population of more than 800 participants using a simplified 2-step task [74].

Overall, human evidence for increased habitual tendencies in AUD is limited, and methodological differences between studies complicate direct comparisons. However, the 2-step studies highlight the predictive power of model-based control for relapse risk and drinking trajectories.

## Discussion

The review highlights that rodent studies consistently show a decrease in goal-directed control and an increase in habitual tendencies after prolonged excessive alcohol experience. Our meta-analysis from more than 400 animals challenges the dichotomous view of habitual and goal-directed responding and provides evidence for a continuum, with chronic alcohol experience shifting the balance towards more habitual responding. Based on the amalgamated findings of published studies, assessing habitual tendencies emerges as a potential indicator of an AUD-like phenotype in animals. Importantly, our meta-analysis offers a framework exemplifying how to address the reproducibility crisis in preclinical research [75, 76], potentially leading to the adoption of more rigorous experimental designs. Ultimately, this may enhance the successful translation of animal findings, fostering a better understanding of human AUD.

The observed response bias in the meta-analysis seems independent of the manner of chronic alcohol experience. Given the substantial differences in experimental protocols regarding alcohol amount, duration, and administration mode, questions arise regarding neuroadaptations associated with habitual responding in these studies, differing quantitatively, qualitatively, or both. While direct comparisons between paradigms are lacking, recent studies shed light. Smith et al. [77]. investigated voluntary alcohol consumption’s effects with or without CIE exposure on brain-wide cFos expression. Regardless of CIE, a history of alcohol drinking induced significant neuroadaptive changes persisting into prolonged abstinence in the PFC and dorsal striatum. CIE and re-access to alcohol compounded the altered cFos response, particularly in the DMS. Additionally, Roland et al. [78]. identified brain regions, notably the dorsal striatum and amygdala, affected by drinking history, showing increased numbers of cFos-positive cells in high drinking compared to low drinking mice. Similarly, Lagström et al. [79]. conducted electrophysiological recordings in brain slices from rats with a 2-month history of intermittent alcohol access versus water-drinking controls. They found enhanced glutamatergic excitability in the DMS, with the opposite effect in the DLS, more pronounced in high compared to low-alcohol drinkers. Neuroadaptations in the DLS returned to control levels after 48 h of abstinence, while the DMS continued to show hyperglutamatergic excitability.

These results suggest varying alcohol exposure or consumption levels can induce similar neuroadaptive changes in the brain, with specific regions showing increased vulnerability to higher doses. Several independent reports implicate the DMS as a critical area, especially sensitive to higher alcohol doses and exhibiting long-term neuroadaptations persisting during prolonged abstinence. Such dose-dependent long-term neuroadaptations in rodents provide insights into the dose-dependent reduction in cognitive control by chronic drinking in humans, as evidenced by analyses of UK Biobank population data [80, 81] and AUD patients [82].

Understanding how chronic alcohol exposure leads to habitual response biases remains a challenge. Numerous aberrant neuroadaptations, culminating in the progressive reprogramming of the striatocortical circuitry, have been documented [83, 84]. This discussion focuses on two crucial pathological mechanisms associated with chronic alcohol exposure: loss of metabotropic glutamate receptor 2 (mGluR2) function and withdrawal-induced neuroinflammation.

Both rodent and human studies have identified a reduction in mGluR2 levels in the mPFC following chronic alcohol exposure [46]. This reduction diminishes long-term plasticity at corticostriatal synapses, leading to impaired executive control and heightened craving [85]. Notably, the mGluR2 deficit affects long-term depression (LTD), a synaptic plasticity form crucial for learning, and may contribute to increased activity of D1-MSN found post-chronic alcohol exposure [21, 83]. Additionally, chronic alcohol enhances output from DLS to substantia nigra pars reticulata and external globus pallidus, suggesting a preference for strengthening the sensorimotor circuit pathway. This disinhibition of DLS output allows for SMC control over behavior, indicating profound functional and structural plasticity alterations in distinct MSN subpopulations that govern reinforcement-related learning.

Another pathological mechanism observed in both humans and rats during early abstinence is progressive neuroinflammation. The microglia-mediated neuroinflammation affects the local diffusion dynamics of neuromodulators [86], potentially contributing to aberrant dopamine level fluctuations observed during protracted abstinence [87]. These fluctuations, characterized by hypo- or hyperdopaminergic states, may serve as vulnerability factors for diminished cognitive control, leading to craving and relapse [88, 89]. Moreover, alcohol-induced neuroinflammation damages white matter tract integrity [90], impairing effective communication in the brain as, for example from the hippocampus to the mPFC [91]. This impedes memory updating processes, such as the extinction of maladaptive memories, thereby decreasing cognitive flexibility.

The discussed alcohol-induced molecular and cellular pathologies, whether specific, as exemplified by mGluR2 alterations, or more general, through neuroinflammatory reactions, may systematically diminish efficiency or speed of communication within the brain. Consequently, less demanding information processing modes may be utilized, resulting in observed biases towards habitual response tendencies. Importantly, these alcohol-induced pathologies are reversible and represent promising targets for novel treatment approaches aimed at enhancing cognitive processing [85, 92]. The potential effects of these interventions on habitual response biases are currently under investigation [93].

Specific stimulus-response associations are encoded by discrete neuron populations known as neuronal ensembles. The existence of ensembles has been demonstrated for alcohol memories using activity-dependent silencing of task-engaged cell populations [94]. Intriguingly, the activity of a specific ensemble in the infralimbic cortex linked to cues signaling drug non-availability could suppress habitual responding for both alcohol and cocaine [95]. This means that even under conditions linked to habitual drug taking and seeking the animal still maintains its ability to regain control over behavior by responding to a different set of cues, and this control is mediated by a discrete set of neurons. Further, functional ensembles are dispersed across the circuitry and form dynamic meta-ensembles (networks of ensembles) encoding information temporarily according to demands thereby allowing efficient and flexible decision-making [96, 97]. The observation by Giannone et al. [39] of overlapping cell populations controlling different behaviors (i.e., instrumental responding and maze navigation) should be explored in the context of dynamic meta-ensembles using recently developed task- or time-specific cellular resolution monitoring techniques [e.g [98, 99]]. Also, the findings that distinct sets of DMS and OFC neurons are active during outcome revaluation and their activity correlates with the degree of goal-directedness but not with habit execution [100], emphasize the need for detailed exploration of the meta-ensembles associated with habitual or goal-directed responding in AUD models. Encouragingly, methods to identify similar types of sparse code in neuronal populations of humans by fMRI are currently being developed [101].

In humans, mixed results have been obtained, but some studies suggest reduced goal-directed control in individuals with AUD, which may be associated with increased relapse risk and alcohol use severity [68, 71, 72]. However, habitual responding in devaluation tests have proven difficult to establish in humans. A potential reason for this discrepancy between human and animal studies could be that the former typically employ secondary rewards such as money or points. Paradigms using oral or intravenous delivery of primary rewards (e.g., juice or alcohol) in human conditioning tasks have been recently established [102, 103]. We suggest adapting these for instrumental responding to improve comparability with animal studies. Additionally, simple motor learning tasks in humans may reveal response biases in AUD subjects, and if so such tasks should be easy to back-translate into animal experiments (e.g., skilled walking task, Fig. 1). Additionally, the 2-step task for assessing model-based versus model-free learning strategies shows promise in predicting drinking behavior or relapse in humans. The successful back-translation of this paradigm to rats and mice [104, 105] will strongly facilitate research on the neurobiological mechanism underlying biased decision-making in AUD.

The relatively modest outcomes of behavioral tasks aimed at uncovering habitual control sharply contrast with the widespread self-description of addictive behaviors, including those related to alcohol, as habitual, whereby the specific interpretation of this term by a subject remains ambiguous. This discrepancy is also evident in weak correlations between self-reports and behavioral measures of the same construct, as observed in patients with substance use disorder assessed with questionnaires evaluating automaticity and devaluation tasks [106]. Similar findings are frequently observed in many fields of experimental psychology, but the underlying reasons for this divergence are not well elucidated [107]. In part, it could be attributed to the disparity between controlled laboratory settings and the complex nature of real-life experiences and may also account for the very limited predictive power (low percentage range) of specific laboratory tasks in predicting alcohol and drug-taking behaviors in humans [106, 108]. Despite the practical challenges associated with their assessment, habitual or automatic response biases have been effectively addressed in the treatment of AUD. Training schedules designed to specifically diminish automatic approach biases towards alcoholic beverages have repeatedly demonstrated effectiveness in enhancing the long-term drinking outcomes of recovery programs [109, 110].

## Conclusions and further directions

How can we integrate habitual response biases into the circle of addiction [11]? A bias towards habitual responding may be particularly important in the protracted withdrawal and anticipation stages, increasing the risk of relapse. However, in the intoxication stage, once a relapse has occurred, mechanisms of compulsivity may be more influential. In this context, we want to stress the distinction between compulsivity and habitual tendencies. Compulsivity is defined as persistent behavior despite adverse consequences, while habitual control is a momentary process that is context and cue-dependent. In animal experiments compulsive responding will persist over a long time or throughout an experimental session and is not strongly influenced by the settings. On the other hand, habitual control is observed only over brief periods after a stimulus and changes to a more adaptive mode often within minutes. Animal studies on alcohol behavior provide only weak support for a direct link between habitual and compulsive control [47, 48]. Although there is some overlap within corticostriatal circuits, compulsive drinking is strongly associated with stress, and emotional regulation, and particularly involves insula circuits [111–113]. Thus, in contrast to common beliefs [12, 13] habitual and compulsive responding are not likely to form a continuum. In our view, habitual biases act as moderators rather than mediators of the relationship between chronic alcohol use and the development of compulsivity. This holds true regardless of whether these biases are pre-existing or acquired through drug use, a question that warrants further investigation in future research.

Taken together, there is limited support for a strict habit-goal dichotomy, particularly in terms of habits being seen as a principal sign of impaired decision-making and loss of control in AUD, or goal-directed behavior being the key to preventing dysfunctional drinking. Indeed, the same behavior, such as animals pressing a lever, can arise from different control systems and potentially different neural circuits. Both goal-directed and automatic decision-making are essential for behavioral flexibility: the automatic system enables quick decisions with minimal cognitive resources, which can be allocated to the goal-directed system when executive control is needed in novel or critical situations. As a result, these two systems may work in parallel and interact in various ways, making it challenging to determine their relative contributions to the control of behavior.

The intricacies of this relationship are inadequately represented by a terminology rooted in a habit-goal dichotomy. Moreover, within the context of addiction the term “habit” carries negative connotations and might exacerbate the stigmatization of individuals affected by it [114]. Instead we suggest adopting a more precise terminology in the context of test paradigms, that describes the probabilistic nature of observed response biases. Phrases like “level of goal-directedness” or “degree of automaticity”, better capture the temporary and dimensional allocation of cognitive resources in complex decision-making processes.

Moving forward, future research should delve into the concepts of model-free and model-based decision-making, especially in rodent models, to address fundamental neurobiological questions about learning, behavioral control, and addiction. Detailed investigations into the molecular and cellular representation of dynamic decision-making, focusing on ensembles and meta-ensembles associated with different degrees of goal-directed responding in AUD models, will offer valuable insights, especially if also considering factors such as sex and age.

On the clinical front, while a lower degree of goal-directedness is consistently observed in reward devaluation tasks in animals with chronic alcohol exposure, the predictive power of similar tests in human studies, including the 2-step task, is limited. Consequently, the utility of these laboratory tests as p clinical markers for AUD severity, progression, or treatment response seems limited. As discussed, individuals with AUD tend to initially rely on less demanding cognitive response strategies, but these systematic biases seem insufficient to fully explain compulsive drug taking. Whether and to what extent individual habitual biases moderate the development of compulsivity remains a question that requires further exploration within theoretical frameworks of addiction.

In conclusion, the available data strongly support the biopsychological model of addiction [115] and a gradual rather than categorical distinction between more goal-directed versus habitual decision-making. External factors including stress may shift this balance. Refining our understanding of decision-making processes and response biases offers promising avenues for both basic research and clinical interventions in AUD.

### Supplementary information

Supplementary Information
