# Supplementary material for: Bad habits–good goals? Meta-analysis and translation of the habit construct to alcoholism
Source: Transl Psychiatry. 2024 Jul 19;14:298. doi: 10.1038/s41398-024-02965-1 (PMC11271507; doi:10.1038/s41398-024-02965-1)
Supplement: Supplementary file 1 — Supplementary Information [file 41398_2024_2965_MOESM1_ESM.docx]

**Supplementary Information**

**Calculation of the effect size**

All experiments included in this meta-analysis adhere to a common design: animals undergo either a satiety-devaluation test or a contingency degradation test, used for testing habits in rodents. Both tests evaluate habitual behavior by comparing a baseline condition (non-devalued – non-degraded) with a test condition (devalued – degraded) within the same set of animals.

Initially, we extracted the mean and the standard error for both baseline and test conditions, across control and ethanol-treated groups, from the provided graphs using WebPlotDigitizer 4.6. Given the sample sizes, we converted standard errors to standard deviations (SD), as explicit means and SDs were not provided in the text.

Considering the repeated nature of the tests, we first calculated the Cohen’s d_av_ as per Cumming and Lakens [1, 2]:

|  | $d_{av}=\frac{M_{diff}}{\frac{{SD}_{1}+{SD}_{2}}{2}}$ | *(1)* |
| --- | --- | --- |

Here, M_diff_ represents the mean difference between the two conditions (baseline and test), and SD_1_ and SD_2_ indicate the standard deviations for the two conditions respectively.

To account for positive bias arising from small sample sizes in the original studies (n < 20), we transformed Cohen’s d_av_ into Hedges’ g_av_ [3]:

|  | $g_{av}=J*d_{av}$ | *(2)* |
| --- | --- | --- |

Where the correction factor *J* is:

|  | $J=1-\frac{3}{4\left( n-1 \right)-1}$ | *(3)* |
| --- | --- | --- |

And *n* is the number of pairs.

In situations where the same animals underwent different tests under different instrumental conditions (e.g., varied schedules or rewards), we calculated a representative effect size. This was achieved by averaging the Hedges’ g_av_ values of each test, ensuring that individual animals were not overrepresented [2, 3].

**Variance estimation and assumptions**

Variance for each effect was calculated based on the formula [3]:

|  | $J^{2}*V_{d}$ | *(4)* |
| --- | --- | --- |

With *J* as the previously mentioned correction factor. The variance of Cohen’s d, *V_d_*, was determined by [3]:

|  | $V_{d}=\left( \frac{1}{n}+\frac{d_{av}^{2}}{2n} \right)2(1-r)$ | *(5)* |
| --- | --- | --- |

Given that none of the studies identified for this meta-analysis reported the correlation coefficient *r*, we opted for assuming r = 0, resulting in the formula:

|  | $V_{d}=\left( \frac{1}{n}+\frac{d_{av}^{2}}{2n} \right)2\left( 1-r \right)=\frac{2}{n}+\frac{d_{av}^{2}}{n}$ | *(6)* |
| --- | --- | --- |

Assuming r = 0 leads to a conservative estimation of the confidence intervals associated to each effect. That is, an overestimation of the variance and a underestimation of the precision.

For calculating the variance associated to the representative effect obtained when combining the effect from different tests performed on the same set of animals, we used the formula [3]:

|  | $V_{d}=\frac{1}{4}(V_{1}+V_{2}+2r\sqrt{V_{1}}\sqrt{V_{2}}$) | *(7)* |
| --- | --- | --- |

Considering the absence of *r* in the individual tests, we assumed r = 1, similarly leading to an overestimation of the variance and a underestimation of the precision [3].

**Rationale and Implications of assumptions**

We used a conservative approach because the primary objective of this meta-analysis is not necessarily to pinpoint the exact magnitude of the effect size of the devaluation/degradation effect but rather to rigorously test the robustness of the observed difference between the two conditions (control and ethanol-treated). By using this conservative variance estimate, we are practically testing the alternative hypothesis (the conditions differ) with a rather stringent test. If an effect is deemed significant under these conditions, the implication is that such a finding is robust and would likely remain significant, if not become more so, under less conservative variance assumptions (that is, by using the true correlation coefficient from the original studies).

**Publication Bias**

The potential for publication bias in our meta-analysis was assessed both statistically, via Egger’s regression test, and graphically, via funnel plot. Egger’s test did not reveal evidence of publication bias in either of the subgroups analyzed (Ctrl: t = .258; p = .800 | EtOH: t = 1.352; p = .196), as visually presented in the funnel plots of the individual subgroups (**Supplementary Figure 1 A)**. However, when considering the entire meta-analysis, the test showed a significant result (Overall: t = 2.668; p = .012), suggesting the presence of potential bias across all experiments. This observation was visually corroborated by the funnel plot (**Supplementary Figure 1 B**). Most of the effects deriving from the control groups appear to cluster in the bottom-left region of the funnel plot, while the majority of the effects derived from the alcohol-treated groups are situated around the top-right. This results in an asymmetrical plot with a gap in the bottom-right area of the funnel, where the imputed effects from the “trim-and-fill” procedure are concentrated.


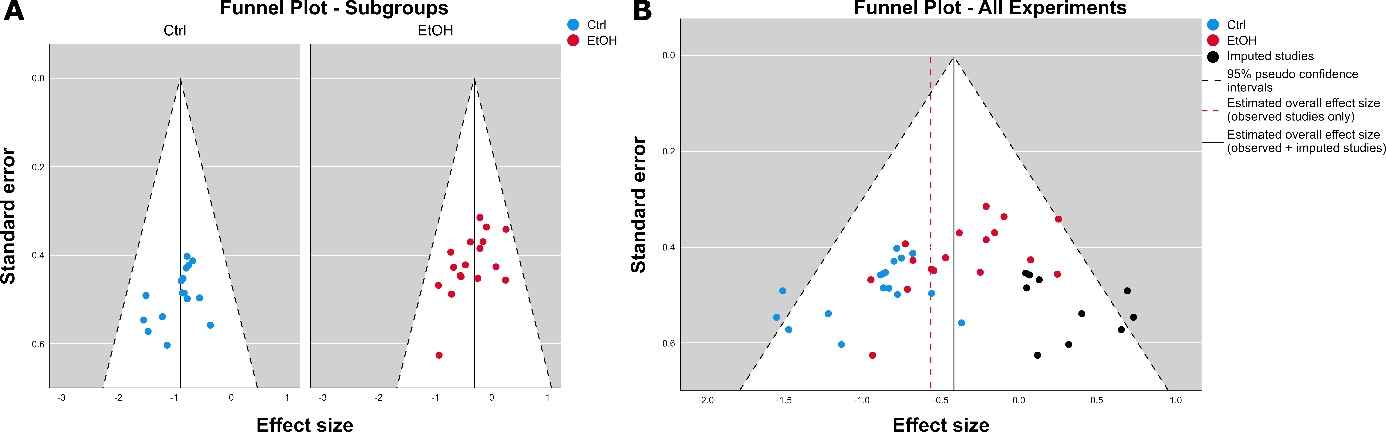


**Supplementary Figure 1.** Funnel plots of the two separate subgroups (A), and of all experiments (B). Effects from the control groups are indicated in blue circles, and effects from the alcohol-treated groups are indicated in red circles. Missing effects imputed by the “trim-and-fill” procedure are indicated in black circles in (B).

It is important to consider the experimental nature of the studies we analyzed in this meta-analysis. Each study utilized either a satiety devaluation followed by an extinction test or a contingency degradation test. In these designs, animals are subjected to a test condition (devalued or degraded) which is compared to a “baseline” condition (non-devalued or non-degraded). A typical outcome is a reduction in lever presses relative to the baseline. A significant reduction indicates goal-directed behavior, whereas a non-significant reduction suggests habitual behavior. Although possible, it’s atypical for lever presses during the test to exceed those from the baseline. Our primary focus was the influence of alcohol on this behavior, hypothesizing that alcohol promotes habitual behavior, manifested as a diminished devaluation/degradation effect. Given these considerations, the asymmetrical shape of the funnel plot–and therefore the significant result of the Egger’s test may reflect the very nature of the effect studied and the influence of alcohol on this effect. Specifically, the hypothesized alcohol effect could result in an aggregation of this subgroup’s effects on the right side of the funnel plot. Since more lever presses during the test compared to baseline are only occasionally observed, alcohol’s influence might create a “flooring effect”, leading to consistently reduced effect sizes and variances as the effects approach their lowest plausible level: the zero-line. This would explain the clustering of the alcohol-treated groups on the top of the funnel plot.

To formally test this hypothesis we conducted a two-way ANOVA, examining the behavior (goal-directed or habitual) and treatment (control or ethanol-treated) influence on the effect sizes’ variance from the studies used in this meta-analysis. Behavior was classified based on the classification reported in the original studies, and the effects that were combined into a representative effect size based on formula *(7)* were used individually in this test. The two-way ANOVA results confirmed our hypothesis, showing a significant effect of Behavior (F_1,46_ = 15.923; p < .001) without any effect of Group (F_1,46_ = 0.554; p = .46), or any significant interaction (F_1,46_ = 0.041; p = .84). The distribution of the variances across the two types of behavior are presented in **Supplementary Figure 2**.


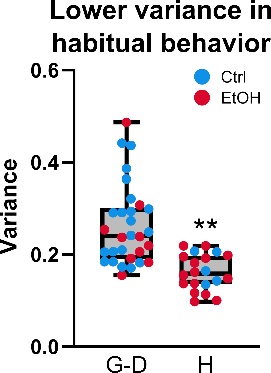


**Supplementary Figure 2.** Box and whisker plot of the variance of the different effect sizes from each individual experiment, aggregated based on behavioral classification: G-D (goal-directed), and H (habitual). Variances associated with the effects of control groups are indicated in blue circles, those associated with the alcohol-treated groups are indicated in red circles.

**Sensitivity Analyses**

To ensure the robustness of our meta-analysis results, we conducted two types of sensitivity analyses.

**1. “Leave-One-Out” Sensitivity Analysis:**

*Rationale.* This approach assesses the influence of individual studies on the overall meta-analytic result. By repeatedly conducting the meta-analysis while excluding one experiment at a time, we can determine if any specific experiment disproportionately impacts the results. We also applied this methodology by excluding the studies that were represented more than once in the meta-analysis (e.g. multiple experiments with independent cohorts within the same study), by removing all sets of experiments deriving from these studies.

*Results.* As detailed in **Supplementary Tables 1** and **2**, the meta-analytic results were consistent across iterations. Regardless of the experiment/study excluded, both subgroups remained significantly different from each other, as well as significantly different from the zero-line.

**2. Correlation Coefficient Sensitivity Analysis:**

*Rationale.* Given that our meta-analysis faced the limitation of lacking correlation coefficients for computing effect sizes and variances for each animal set, we explored the impact of this uncertainty by recalculating effect sizes and variances using different correlation values *r*.

*Methodology.* Effect sizes for each group were calculated as Cohen’s d_z_ [2] using formula *(8)*, and variances were determined based on formula *(5)*. We tested different values of *r* including 0.1, 0.3, 0.5, 0.7, and 0.9. For combined representative effects derived from multiple tests on the same animal set in different instrumental conditions, we still assumed *r* = 1 as in our original approach, based on formula *(7),* since this is the most conservative approach.

*Results.* The sensitivity analysis, displayed in **Supplementary Table 3**, revealed consistent results for all *r* values. In all iterations, the two subgroups were statistically different from each other, and both differed significantly from the zero-line. This confirms that our conservative approach and the resulting outcomes are robust.

|  | $d_{z}=\frac{M_{diff}}{S_{diff}}$ | *(8)* |
| --- | --- | --- |

Where *S_diff_* is

|  | $\sqrt{{SD}_{1}^{2}+{SD}_{2}^{2}-2*r*{SD}_{1}*{SD}_{2}}$ | *(9)* |
| --- | --- | --- |

| **Classification** | | **Meta-Analysis** | | | | **Egger's test** |
| --- | --- | --- | --- | --- | --- | --- |
| **Excluded experiment** | **Subgroup** | **ES (d_av_)** | **CI lower** | **CI upper** | **p-value** | **p-value** |
| Corbit LH. 2012 | Ctrl | -0.91 | -1.15 | -0.68 | 0.00 | .780 |
|  | EtOH | -0.33 | -0.53 | -0.12 | 0.00 | .215 |
|  | All | -0.58 | -0.75 | -0.42 | 0.00 | .017 |
| Fisher H. 2017 | Ctrl | -0.91 | -1.15 | -0.67 | 0.00 | .800 |
|  | EtOH | -0.28 | -0.48 | -0.09 | 0.00 | .277 |
|  | All | -0.55 | -0.71 | -0.39 | 0.00 | .014 |
| Barker JM. 2017 (1) | Ctrl | -0.93 | -1.16 | -0.69 | 0.00 | .761 |
|  | EtOH | -0.34 | -0.54 | -0.14 | 0.00 | 133 |
|  | All | -0.59 | -0.75 | -0.43 | 0.00 | .009 |
| Barker JM. 2017 (2) | Ctrl | -0.90 | -1.13 | -0.67 | 0.00 | .769 |
|  | EtOH | -0.30 | -0.49 | -0.10 | 0.00 | .263 |
|  | All | -0.55 | -0.71 | -0.40 | 0.00 | .010 |
| Barker JM. 2017 (3) | Ctrl | -0.91 | -1.14 | -0.67 | 0.00 | .799 |
|  | EtOH | -0.30 | -0.50 | -0.10 | 0.00 | .212 |
|  | All | -0.56 | -0.72 | -0.40 | 0.00 | .013 |
| Barker JM. 2017 (4) | Ctrl | -0.91 | -1.15 | -0.68 | 0.00 | .799 |
|  | EtOH | -0.30 | -0.50 | -0.11 | 0.00 | .212 |
|  | All | -0.57 | -0.73 | -0.41 | 0.00 | .013 |
| Renteria R. 2018 | Ctrl | -0.92 | -1.16 | -0.64 | 0.00 | .797 |
|  | EtOH | -0.32 | -0.53 | -0.12 | 0.00 | .201 |
|  | All | -0.58 | -0.75 | -0.42 | 0.00 | .013 |
| Renteria R. 2020 | Ctrl | -0.88 | -1.11 | -0.64 | 0.00 | .765 |
|  | EtOH | -0.36 | -0.57 | -0.16 | 0.00 | .166 |
|  | All | -0.58 | -0.74 | -0.43 | 0.00 | .077 |
| Barker JM. 2020 (1) | Ctrl | -0.89 | -1.12 | -0.66 | 0.00 | .860 |
|  | EtOH | -0.34 | -0.53 | -0.14 | 0.00 | .179 |
|  | All | -0.57 | -0.73 | -0.42 | 0.00 | .019 |
| Barker JM. 2020 (2) | Ctrl | -0.93 | -1.16 | -0.70 | 0.00 | .514 |
|  | EtOH | -0.32 | -0.51 | -0.12 | 0.00 | .181 |
|  | All | -0.59 | -0.75 | -0.43 | 0.00 | .007 |
| Cazares C. 2021 | Ctrl | -0.87 | -1.10 | -0.64 | 0.00 | .836 |
|  | EtOH | -0.33 | -0.54 | -0.13 | 0.00 | .237 |
|  | All | -0.56 | -0.72 | -0.41 | 0.00 | .030 |
| Towner TT. 2021 (1) | Ctrl | -0.93 | -1.16 | -0.69 | 0.00 | .866 |
|  | EtOH | -0.29 | -0.49 | -0.09 | 0.00 | .212 |
|  | All | -0.57 | -0.73 | -0.40 | 0.00 | .011 |
| Towner TT. 2021 (2) | Ctrl | -0.91 | -1.15 | -0.68 | 0.00 | .802 |
|  | EtOH | -0.31 | -0.51 | -0.11 | 0.00 | .180 |
|  | All | -0.57 | -0.74 | -0.41 | 0.00 | .012 |
| Towner TT. 2021 (3) | Ctrl | -0.91 | -1.14 | -0.67 | 0.00 | .796 |
|  | EtOH | -0.29 | -0.48 | -0.09 | 0.01 | .173 |
|  | All | -0.56 | -0.72 | -0.39 | 0.00 | .010 |
| Towner TT. 2021 (4) | Ctrl | -0.91 | -1.14 | -0.67 | 0.00 | .799 |
|  | EtOH | -0.31 | -0.52 | -0.12 | 0.00 | .205 |
|  | All | -0.58 | -0.74 | -0.42 | 0.00 | .016 |
| Ma T. 2022 | Ctrl | -0.92 | -1.16 | -0.68 | 0.00 | .822 |
|  | EtOH | -0.30 | -0.50 | -0.10 | 0.00 | .211 |
|  | All | -0.57 | -0.73 | -0.41 | 0.00 | .011 |
| Giannone F. 2022 | Ctrl | -0.88 | -1.11 | -0.65 | 0.00 | .981 |
|  | EtOH | -0.30 | -0.49 | -0.10 | 0.00 | .240 |
|  | All | -0.55 | -0.70 | -0.39 | 0.00 | .021 |

**Supplementary Table 1. “Leave-One-Out” sensitivity analysis for each individual experiment.** The meta-analysis was performed repeatedly by excluding one experiment each time (the excluded experiments are indicated in the left column).

| **Classification** | | **Meta-Analysis** | | | | **Egger's test** |
| --- | --- | --- | --- | --- | --- | --- |
| **Excluded study** | **Subgroup** | **ES (d_av_)** | **CI lower** | **CI upper** | **p-value** | **p-value** |
| Barker JM. 2017 | Ctrl | -0.93 | -1.18 | -0.67 | 0.00 | .690 |
|  | EtOH | -0.30 | -0.51 | -0.09 | 0.00 | .175 |
|  | All | -0.57 | -0.75 | -0.39 | 0.00 | .008 |
| Barker JM. 2020 | Ctrl | -0.92 | -1.15 | -0.68 | 0.00 | .548 |
|  | EtOH | -0.34 | -0.54 | -0.14 | 0.00 | .162 |
|  | All | -0.59 | -0.75 | -0.43 | 0.00 | .010 |
| Towner TT. 2021 | Ctrl | -0.95 | -1.22 | -0.68 | 0.00 | .875 |
|  | EtOH | -0.25 | -0.48 | -0.03 | 0.03 | .170 |
|  | All | -0.57 | -0.76 | -0.37 | 0.00 | .011 |

**Supplementary Table 2. “Leave-One-Out” sensitivity analysis for over-represented studies.** The meta-analysis was performed repeatedly by excluding those studies that were represented more than once in the meta-analysis due to multiple independent experiments within the same study (the excluded studies are indicated in the left column).

| **Correlation coefficient *r*** | **Overall d_z_ [CIs]** | **Ctrl group d_z_ [CIs]** | **EtOH group d_z_ [CIs]** | **Subgroups different from each other?** | **Subgroups different from Zero-Line?** |
| --- | --- | --- | --- | --- | --- |
| 0.1 | -0.42 [-0.55; -0.29] | -0.64 [-0.83; -0.44] | -0.23 [-0.41; -0.06] | Yes | Yes (Both) |
| 0.3 | -0.45 [-0.57; -0.34] | -0.70 [-0.88; -0.52] | -0.26 [-0.42; -0.10] | Yes | Yes (Both) |
| 0.5 | -0.53 [-0.66; -0.40] | -0.78 [-0.94; -0.63] | -0.30 [ -0.45; -0.14] | Yes | Yes (Both) |
| 0.7 | -0.65 [ -0.80; -0.49] | -0.91 [-1.04; -0.79] | -0.37 [-0.56; -0.19] | Yes | Yes (Both) |
| 0.9 | -0.93 [-1.17; -0.69] | -1.32 [-1.61; -1.02] | -0.54 [-8177; -0.26] | Yes | Yes (Both) |

**Supplementary Table 3. Correlation coefficient sensitivity analysis.** The meta-analysis was performed repeatedly by assuming each time a different correlation coefficient *r* (left column) for calculating both the effect size (Cohen’s d_z_) and the confidence intervals (CIs).

**References**

1. Cumming, G., *Understanding the new statistics: Effect sizes, confidence intervals, and meta-analysis*. Understanding The New Statistics: Effect Sizes, Confidence Intervals, and Meta-Analysis. 2012, New York, NY, US: Routledge/Taylor & Francis Group. xiv, 519-xiv, 519.

2. Lakens, D., *Calculating and reporting effect sizes to facilitate cumulative science: a practical primer for t-tests and ANOVAs.* Frontiers in Psychology, 2013. **4**: p. 863.

3. Borenstein, M., Cooper, H., Hedges, L., and Valentine, J., *Effect sizes for continuous data.* The Handbook of Research Synthesis and Meta-Analysis, 2009. **2**: p. 221-235.
